# Neglected giant bladder stone with bilateral ureteral stones: A case report of staged surgical treatment

**DOI:** 10.1016/j.ijscr.2025.110933

**Published:** 2025-02-04

**Authors:** Iwan Purnomo Aji, Johan Renaldo, Dimas Panca Andhika

**Affiliations:** a Department of Urology, Faculty of Medicine, Universitas Airlangga, Dr. Soetomo Academic General Hospital, Surabaya, Indonesia; b Department of Urology, Universitas Airlangga Teaching Hospital, Surabaya, Indonesia

**Keywords:** Giant bladder stone, Ureteral stone, Hydronephrosis, Vesicolithotomy, Ureteroscopy, Staged surgery

## Abstract

**Introduction and importance:**

A giant bladder stone is a rare urological condition in which a massive stone forms due to various contributing factors. We present a rare case of a giant bladder stone with concurrent ureteral stones, detailing its staged surgical management and relevant literature.

**Case presentation:**

A 44-year-old male presented with right flank, left flank, and suprapubic pain for one month prior to admission, accompanied by dysuria and a history of stones passage through the urinary tract. On examination, the patient presented with suprapubic pain, and laboratory results revealed severe anemia with elevated blood urea nitrogen (BUN) and serum creatinine levels. A vesicolithotomy was performed, followed by ureteroscopic lithotripsy (URS) one month later. A 15 × 10 cm bladder stone was found during the first surgery, and multiple ureteral stones were discovered during the second surgery. After surgery, the patient improved BUN (93.5 mg/dL to 27.6 mg/dL), serum creatinine (8.11 mg/dL to 1.85 mg/dL), and reduced flank and suprapubic pain.

**Clinical discussion:**

The management of giant bladder stones involves open vesicolithotomy, which is considered the gold standard for complete removal, as AUA and EAU guidelines recommend. A subsequent URS for the removal of bilateral ureteral stones provides a favorable outcome for the patient.

**Conclusion:**

A holistic approach for giant bladder stones is required, encompassing diagnosis and surgical planning to minimize misdiagnosis and complications. A staged surgical approach, including vesicolithotomy and ureteroscopic lithotripsy, may be beneficial.

## Highlights

• An adult male presenting with urinary symptoms was diagnosed with a giant bladder stone and bilateral ureteral stones.
• Imaging studies revealed multiple stones, including a large bladder stone and several ureteral stones.
• The patient underwent staged surgical interventions, successfully addressing the stones and alleviating symptoms.

## 1 Introduction

Bladder stones account for only 5 % of all urinary tract stones [1]. A giant bladder stone is a rare condition defined as a stone measuring >4 cm in diameter and weighing >100 g, with fewer than 100 reported cases, according to recent literature [2,3]. Some studies indicate that giant bladder stones are more frequently reported in developing and tropical countries [4]. A giant bladder stone presenting with concurrent ureteral stone is even rarer, with limited published research available. This condition poses a significant challenge due to its rarity, large size, and potential to cause complex symptoms and serious complications, often necessitating more invasive medical interventions [5].

The primary treatment for managing giant bladder stones remains open vesicolithotomy due to its high success rate and its ability to remove large stones in a single operation [5,6]. The treatment of choice for ureteral stone depends on the stone size, with ureteroscopy (URS) preferred for stones measuring >10 mm [7].

This case report presents a rare instance of a giant bladder stone with bilateral ureteral stones and discusses the potential complications and outcomes associated with a staged surgical approach. This report is presented in accordance with the SCARE and PROCESS guidelines [8,9].

## 2 Case presentation

A 44-year-old male patient presented with a chief complaint of pain in the right flank, left flank, and suprapubic regions, with the most severe pain in the left waist that had been present for one month. The patient also complained of a burning sensation during urination for the past two years, which had not been treated. The patient also reported passing a stone from the urinary tract the size of a grain of sugar two weeks prior. No history of hematuria or lower urinary tract symptoms was reported. There was suprapubic tenderness without flank mass or costovertebral angle tenderness. Urine production was 500 mL/24 h, with yellow color.

**Fig. 1:**
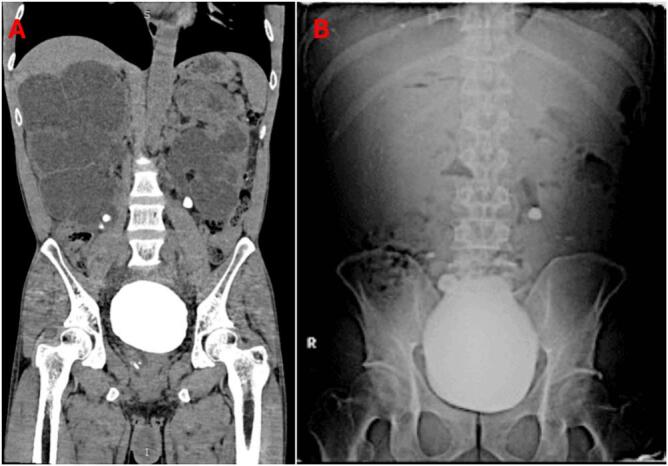
(A) CT sonography and (B) Abdominal plain radiograph.

**Fig. 2:**
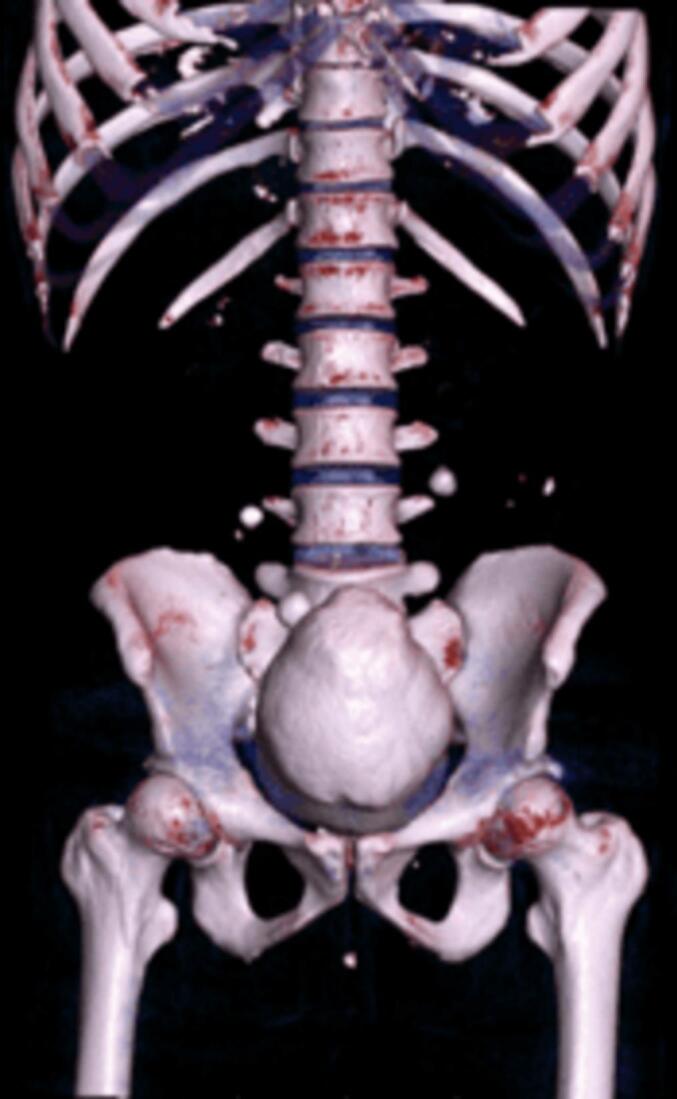
3D CT scan.

Laboratory tests revealed a hemoglobin level of 7.9 g/dL and elevated BUN and serum creatinine levels at 93.5 and 8.11 mg/dL, respectively. The patient underwent plain abdominal X-rays and a CT sonography. The abdominal X-ray showed four radiopaque shadows: a 7 mm stone at the level of the proper L4-L5 vertebrae, a 17 × 18 mm stone at the level of the proper L5-S1 vertebrae, a 12 × 11 mm stone at the level of the left L3-L4 vertebrae, and a large 10 × 12 cm stone occupying the pelvic cavity (Fig. 1). The CT sonography revealed a large bladder stone measuring 10.9 × 8.5 × 11.5 cm with a density of 1384 Hounsfield units (HU). Additionally, the right kidney showed severe hydronephrosis and hydroureter, with a 0.5 × 1 cm stone measuring 838 HU in the lower pole and a 1.6 × 1.6 cm stone with a density of 1557 HU in the medial ureter. The left kidney also had severe hydronephrosis and hydroureter, with two stones in the lower pole measuring 0.3 cm and 0.4 cm with densities of 381 HU, as well as a larger stone in the proximal ureter measuring 1.4 × 1.1 cm and 1765 HU (Figs. 1, 2).

**Fig. 3:**
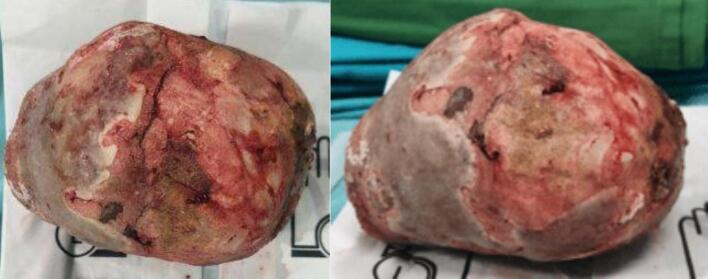
A 15 × 10 cm bladder stone was extracted via vesicolithotomy, emphasizing the necessity of open surgical intervention due to its significant size.

Two units of packed red cell transfusion were given before surgery to manage the anemia. The patient then underwent vesicolithotomy and nephrostomy placement in both the right and left kidneys as temporary urinary diversion using a 16 Fr silicone catheter. The large bladder stone was successfully removed, measuring 15 × 10 cm (Fig. 3). A cystostomy was also placed for drainage. The procedure was done by an experienced senior urologist.

**Fig. 4:**
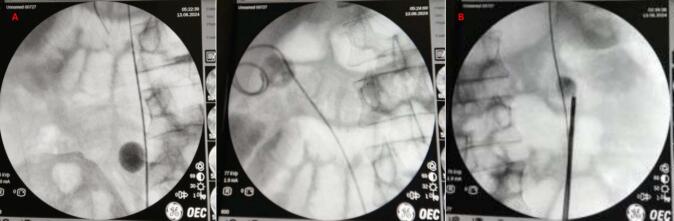
(A) Right ureteroscopy (URS) demonstrating a 1.6 × 1.6 cm stone, successfully managed with double-J (DJ) stent placement to relieve obstruction and ensure urinary drainage. (B) Left ureteroscopy (URS) identified a 1.4 × 1.1 cm stone, effectively fragmented using lithotripsy; however, attempts to place a DJ stent were unsuccessful.

The patient underwent a second surgery one month later. Ureteroscopy (URS) was performed on both sides. URS was chosen because it is effective for treating stones that are unlikely to respond to ESWL due to high density (>1000 HU), large size, and complex locations. It allows direct visualization, precise laser fragmentation, and the ability to treat bilateral stones in a single session, reducing the need for repeated procedures and achieving higher stone-free rates. A 1.6 × 1.6 cm stone was found in the right URS, and a DJ stent was placed (Fig. 4). A 1.4 × 1.1 cm stone was found in the left URS, and URS lithotripsy was performed, successfully fragmenting the stone. However, the placement of a DJ stent on the left side failed as the stent could not be advanced through the ureter. Finally, an 18 Fr urethral catheter was placed, and the right nephrostomy was removed.

**Fig. 5:**
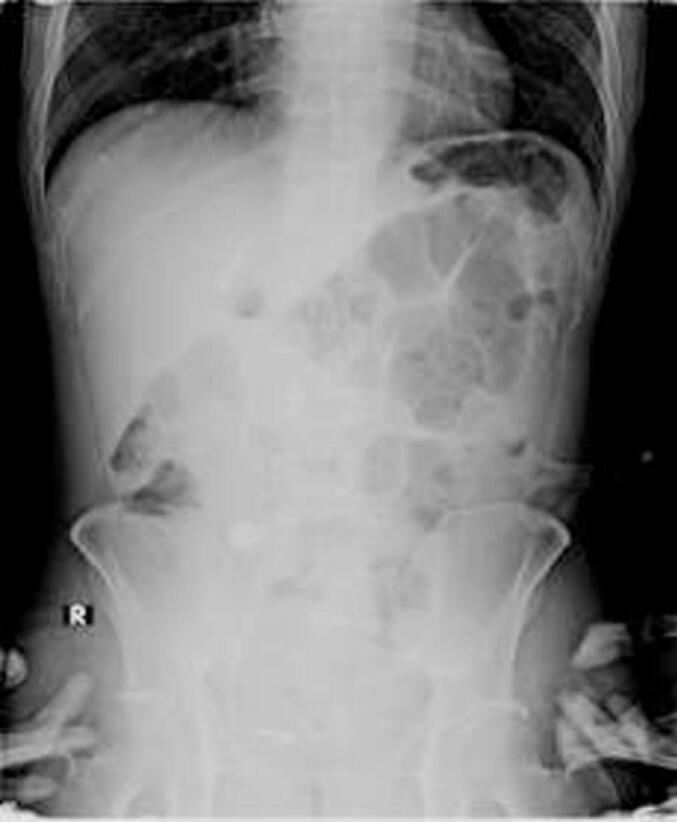
Postoperative abdominal plain radiograph: The proximal tip of the DJ stent is located near the right L1 vertebra, while the distal tip is in the pelvic cavity. A radiopaque oval-shaped shadow is observed near the right L5 vertebra, consistent with a right ureteral stone.

After surgery, the patient reported reduced pain in the flank and suprapubic regions. Urine output increased to 1000 mL/16 h with a clear yellow color. Laboratory tests showed improved BUN and serum creatinine levels at 27.6 and 1.85 mg/dL, respectively. A plain abdominal X-ray showed an oval-shaped radiopaque shadow at the correct L5 vertebra, suggesting the residual right ureteral stone (Fig. 5). The patient remains with a left nephrostomy and cystostomy in place.

The proposed next step for this patient is right URS lithotripsy to fragment the remaining ureteral stone.

## 3 Discussion

Bladder stones account for 5 % of all urinary tract stone cases. Bladder stones are more common in developing countries, with a male-to-female ratio ranging from 10:1 to 4:1. The prevalence of urinary tract stones ranges between 5 % and 19.1 % in the Asian population [1,10,11]. Large bladder stones with a >4 cm diameter and weighing >100 g are rarely found in modern urology cases [4,6,12]. So far, fewer than 100 cases of large bladder stones have been reported in the English-language medical literature, with the largest stone weighing 1640 g [12]. Bladder outlet obstruction, genetic or metabolic diseases, and various environmental factors such as hormonal issues, climate, lifestyle, socioeconomic status, and education level can be identified as contributing factors [1,13]. Dietary factors and socioeconomic status also play an important role in forming large bladder stones [6]. The risk factors we identified in our patient are being male, 44 years old, living in a tropical area, being of Asian race, and residing in Indonesia.

The size of the stone is the main factor to consider when determining the therapeutic modality for large bladder stones. Current guidelines for bladder stones still rely on the linear measurement of total stone diameter to categorize stones into <5 mm, 5–10 mm, 10–20 mm, and >20 mm [7]. Due to their large size, the gold standard intervention that can be performed is open vesicolithotomy, which allows for complete stone removal. Almost all cases of large bladder stones are managed with vesicolithotomy, with promising success rates [1,4,12,14]. This approach is also strongly recommended for large bladder stones by the American Urological Association (AUA), with a grade C recommendation [15], and also by The European Association of Urology (EAU) [7]. Although this intervention has proven highly effective, it is important to recognize that it requires catheterization and prolonged hospitalization [7,10].

While effective for stones smaller than 2 cm, endourological approaches such as transurethral lithotripsy are challenging to perform on larger bladder stones [1]. Due to the large dimension of the stones and the fact that most patients have accompanying urinary tract infections, the efficiency of transurethral lithotripsy is low. Additionally, transurethral lithotripsy increases the risk of infection and has a longer operating time, which increases the incidence of postoperative complications [4].

The choice of ureteral stone removal procedure is based on factors such as stone size, location, and density. For ureteral stones, about 95 % of stones up to 4 mm in size are expected to pass spontaneously within 40 days, along with 71 % of stones <5 mm in size in the proximal ureter and 89 % of stones of the same size in the distal ureter. However, this percentage decreases as the size of the stone increases [16]. Larger stones achieve a stone-free condition faster with URS, the preferred treatment for stones measuring 10 mm or more in diameter [7]. Stone density is also a factor, with URS more preferable in high-density (>1000 HU) stones, allowing direct visualization, precise laser fragmentation, and the ability to treat bilateral stones in a single session, reducing the need for repeated procedures [17].

The staged surgical approach, consisting of vesicolithotomy followed by URS, provided an effective solution for managing the giant bladder stone (15 × 10 cm) and bilateral ureteral stones in this case. The main advantage of open vesicolithotomy is its high success rate for complete stone removal in a single operation, particularly for giant stones. This is supported by Dursun et al., who reported that elderly patients with larger bladder stones benefitted significantly from open surgery due to the complexity of their cases and associated comorbidities [18]. Additionally, the staged nature of the procedure allowed for recovery from severe anemia before addressing the ureteral stones, reducing the risk of perioperative complications. Furthermore, Duarsa et al. demonstrated the effectiveness of staged approaches in cases involving multiple stones, highlighting the benefit of sequential surgeries in reducing overall surgical stress and improving outcomes [19].

However, the approach has notable drawbacks. Open vesicolithotomy requires longer hospitalization, surgical scars, and a potentially extended recovery period compared to minimally invasive techniques. Cancian et al. emphasized that combining open and endoscopic approaches can balance invasiveness and effectiveness for large bladder stones and anatomically complex cases. However, advanced tools and expertise are often required [20]. However, minimally invasive methods are limited by their inefficacy in managing bladder stones larger than 2 cm, as noted by Fauzi et al., particularly in resource-limited settings where advanced technology like holmium laser lithotripsy may not be available. In this case, ureteroscopy lithotripsy was determined to be the most suitable intervention, as it allows for precise fragmentation and removal of bilateral ureteral stones while minimizing patient morbidity. The placement of a double-J (DJ) stent is a critical adjunct to ensure optimal urinary drainage following the procedure, thereby mitigating the risk of postoperative complications such as ureteral obstruction, hydronephrosis, or infection. Additionally, URS was selected based on its proven efficacy in managing urinary calculi that are unlikely to respond to extracorporeal shock wave lithotripsy (ESWL), particularly in cases involving stones with high attenuation values exceeding 1000 HU, substantial stone size, or anatomically complex locations [21].

Comparative outcomes reinforce the staged approach's practicality in resource-limited environments. Fauzi et al. noted that large bladder stones often necessitate open surgery due to the increased risk of incomplete fragmentation and complications with minimally invasive techniques [21]. In this case, the staged approach resulted in significant symptomatic relief, improved renal function, and successful resolution of the giant bladder and ureteral stones. The balance between resource availability, stone size, and patient condition ultimately dictated the choice of the staged surgical strategy, validating its utility in complex cases.

## 4 Conclusion

In cases of a giant bladder stone with concurrent ureteral stones, a holistic approach is essential, encompassing the diagnostic process and surgical planning to minimize diagnostic errors as well as intraoperative and postoperative complications. A staged surgical approach with vesicolithotomy and URS provided a favorable outcome for our patient, resulting in significant clinical and renal function improvement. Additionally, clinicians must anticipate and manage potential complications or conditions that may arise before and after surgery.

## CRediT authorship contribution statement

Iwan Purnomo Aji: Conceptualization, Writing - original draft, Writing - review & editing, Investigation.

Johan Renaldo: Conceptualization, Supervision, Methodology, Writing - original draft, Writing - review & editing, Validation.

Dimas Panca Andhika: Conceptualization, Resources, Supervision, Methodology, Project Administration, Writing - original draft.

## Consent

The patient provided written informed consent to publish this case report and accompanying images. The editor-in-chief of this journal can review a copy of the written consent upon request.

## Ethical approval

The hospital research ethics committee, where the patient was admitted, gave ethical approval for reporting this case.

## Guarantor

1. Johan Renaldo
2. Dimas Panca Andhika

## Research Registration Number

NA.

## Provenance and peer review

Not commissioned, externally peer-reviewed.

## Provenance and peer review

Not commissioned, externally peer-reviewed.

## Sources of funding

The authors received no financial support for this article's research, authorship, and/or publication.

## Declaration of competing interest

The authors report no declarations of interest.
